# *Erwinia wuhanensis* sp. nov. isolated from human blood

**DOI:** 10.3389/fmicb.2025.1675452

**Published:** 2025-09-04

**Authors:** Yingmiao Zhang, Yu Zhan, Jing Yang, Zhongxin Lu

**Affiliations:** ^1^Department of Medical Laboratory, The Central Hospital of Wuhan, Tongji Medical College, Huazhong University of Science and Technology, Wuhan, China; ^2^Hubei Provincial Engineering Research Center of Intestinal Microecological Diagnostics, Therapeutics, and Clinical Translation, Wuhan, China; ^3^National Key Laboratory of Intelligent Tracking and Forecasting for Infectious Diseases, Chinese Center for Disease Control and Prevention, Beijing, China; ^4^Cancer Research Institute of Wuhan, The Central Hospital of Wuhan, Tongji Medical College, Huazhong University of Science and Technology, Wuhan, China

**Keywords:** *Erwinia wuhanensis*, novel species, bacteria, genome, 16S rRNA

## Abstract

**Introduction:**

A novel *Erwinia* strain, BC051422^T^, was isolated from the blood of a patient at the Central Hospital of Wuhan, Wuhan, PR China, in 2022. The strain was identified as gram-negative, facultatively anaerobic, motile, and rod-shaped.

**Methods:**

Preliminary analysis based on the 16S rRNA gene sequence and multilocus sequence analysis of the *atpD, infB, rpoB*, and *gyrB* genes unveiled that this strain is closely related to *Erwinia* members. Whole-genome sequencing was performed, and the average nucleotide identity (ANI) and *in silico* DNA–DNA hybridization (isDDH) values between strain BC051422^T^ and type strains of all known *Erwiniaceae* species ranged from 68.8% to 83.4% and 19.2% to 35.5%, respectively, which below the accepted species delineation thresholds of 95% for ANI and 70% for isDDH. The major cellular fatty acids of strain BC051422^T^ were C_16:0_, C_16:1_ ω7c/C_16:1_ ω6c, and C_17:0_ cyclo, consistent with profiles similar to those observed in other *Erwinia* species. The genomic DNA G+C content was 55.95 mol%. Strain BC051422^T^ can be differentiated from other *Erwinia* species by its ability to ferment sucrose, but its inability to metabolize mannose, rhamnose, melibiose, and sorbitol.

**Results:**

Genotypic and phenotypic characteristics together support the classification of strain BC051422^T^ as a novel species of the genus *Erwinia*, for which the name *Erwinia wuhanensis* sp. nov. is proposed. The type strain of *E. wuhanensis* sp. nov. is BC051422^T^ (=GDMCC 1.4074^T^ = JCM 36319^T^).

## Introduction

The genus *Erwinia* was first described by (Winslow et al. 1920) in 1920 and is currently classified under the family *Erwiniaceae*, which was proposed to refine the taxonomy within the order Enterobacterales (Adeolu et al., 2016). According to the List of Prokaryotic Names with Standing in Nomenclature (https://lpsn.dsmz.de/genus/erwinia) (Parte et al., 2020), 20 *Erwinia* species have been validly published to date. Members of the *Erwinia* genus are widely distributed and primarily isolated from plants, including fruit trees, potato stems, pomelo, and olive (Geider et al., 2006; Ming-Kai et al., 2022; Moretti et al., 2011; Ramirez-Bahena et al., 2016). Some species, such as *E. aphidicola, E. typographi*, and *E. teleogrylli*, have been isolated from insects like pea aphid, bark beetles, and crickets, respectively (Harada et al., 1997; Liu et al., 2016; Skrodenyte-Arbaciauskiene et al., 2012). Several *Erwinia* species are known plant pathogens. *E. pyrifoliae, E. piriflorinigrans, E. uzenensis*, and *E. amylovora* are causative agents of fire blight and blossom necrosis in pome fruit trees (Kharadi et al., 2019; Kim et al., 1999; López et al., 2011; Matsuura et al., 2012). A recently described phytopathogen, *E. sorbitola*, has demonstrated potential pathogenicity to both plants and animals (Tao et al., 2023). Despite this, human infections caused by *Erwinia* species remain rare. *E. persicina* CDC 4073-83 was isolated from the urine of an elderly woman with a urinary tract infection and also from bile fluid cultures of another patient with perihilar cholangiocarcinoma (Ceylan and Özden, 2022; O'Hara et al., 1998). *E. billingiae* has been implicated in cutaneous infections, bacteremia, and septic arthritis, with cases linked to environmental exposure to plants (Bonnet et al., 2019; Prod'homme et al., 2017). *E. tasmaniensis* was recovered from the severely necrotic tissue of a patient with cervical lymphadenitis and reported as an *Erwinia*-like organism (Shin et al., 2008). In this study, we report the taxonomic characterization of strain BC051422^T^, isolated from the blood of a patient with chronic renal failure following hemodialysis. Comprehensive genomic and phenotypic analyses indicate that this strain represents a novel species of the genus *Erwinia*, for which we propose the name *Erwinia wuhanensis* sp. nov.

## Materials and methods

### Isolation and ecology

Strain BC051422^T^ was recovered from the blood culture of a 64-year-old female patient presenting with the symptom of fever after hemodialysis at the Central Hospital of Wuhan (Wuhan; 30 °35′ N, 114 °19′ E; PR China) in 2022. The patient developed fever 2 h after regular hemodialysis, with a body temperature of 38.3 °C. After the consultation, the patient was found to have a history of hypertension chronic heart failure, chronic renal failure, maintenance hemodialysis, and renal anemia. She also had a history of type 2 diabetes for more than 10 years, diabetic nephropathy, diabetic retinopathy, fatty liver, gallstones, gastric ulcers, and had undergone appendectomy and amputation of both hands. Laboratory tests revealed a white blood cell (WBC) count of 12.0 × 10^9^/L (94.1% neutrophils) (normal 3.59.5 × 10^9^/L, 40%−75% neutrophils), hemoglobin of 102 g/L (normal 115–150 g/L), and C-reactive protein of 4.54 mg/dL (normal 0–0.6 mg/dL), and procalcitonin of 3.98ng/mL (normal 0–0.05 ng/mL). The patient tested negative for the novel coronavirus. A computerized tomography scan of Lung and abdomen showed bronchitis, multiple small nodules in both lungs, splenomegaly, bilateral kidney atrophy and stones, proximal ureteral stones on the right side, and sclerosis of the abdominal aorta and its branches and bilateral renal blood vessels. Due to the incresed indicators of inflamation, two sets of blood culture were performed. One of the cultures was positive for gram-negative rods after 18 h of incubation at 37 °C in the presence of 5% CO_2_ on Columbia blood agar and MacConkey agar plates (Guangzhou Dijing Microbial Technology Co., Ltd., Guangzhou, China). The antimicrobial susceptibility of strain BC051422^T^ was conducted on VITEK 2 platform (bioMérieux) according to the manufacturer's guidelines, and drug sensitivity was determined according to the Clinical and Laboratory Standards Institute standard (Supplementary Table S1). The isolate is sensitive to all antibiotics tested. The patient's condition improved after receiving piperacillin-tazobactam sodium (4.5 g/12 h) and discharged 6 days after admission.

### 16S rRNA gene sequence analysis

The genomic DNA of purified bacteria was extracted using the TIANamp Bacteria DNA Kit (TIANGEN Biotech, Co., Ltd, Beijing, China). 16S rRNA gene sequencing was performed with universal primers (27F: 5′-AGTTTGATCMTGGCTCAG-3′; 1492R: 5′-GGTTACCTTGTTACGACTT-3′). The amplification was performed on the C1000 Thermal Cycler (Bio-Rad Laboratories, Hercules, CA, USA), and the product was sequenced on the Applied Biosystems 3730XL platform (Thermo Fisher Scientific Inc., MA, USA). The nearly complete 16S rRNA sequence of the strain BC051422^T^ was analyzed with reference to the EzBioCloud Database (Yoon et al., 2017a). The phylogenetic tree was constructed using the neighbor-joining (NJ) and maximum-likelihood (ML) algorithms with Kimura's two-parameter model as well as maximum parsimony algorithm by using the MEGA software version 11 (Tamura et al., 2021).

### Multilocus sequence analysis

The internal sequence fragments of partial *atpD, infB, rpoB*, and *gyrB* of strain BC051422^T^ were retrieved from their whole-genome sequences. Phylogenetic tree based on concatenated partial *atpD, infB, rpoB*, and *gyrB* sequences was constructed using the PhyloSuite platform (Zhang et al., 2020). The best partitioning scheme and evolutionary models were selected using PartitionFinder2 v2.1.1 (Lanfear et al., 2017), with the rcluster algorithm and AICc criterion. Bayesian Inference phylogenies were inferred using MrBayes v3.2.7 (Ronquist et al., 2012) under the partition model (2 parallel runs, 200,000 generations), wherein the initial 25% of the sampled data were discarded as burn-in.

### Genome analysis

The draft genome sequencing of the strain BC051422^T^ was performed using the Illumina HiSeq platform by generating paired-end libraries. Fragmented genomic DNA with an average size of 300 bp was selected for sequencing. The raw data of sequencing were evaluated by FastQC v0.11.2 and cut by Trimmomatic v0.36 (Bolger et al., 2014) to obtain relatively accurate and effective data. The filtered reads were assembled into contigs using SPAdes v3.5.0 (Bankevich et al., 2012), and GapFiller v1.11 (Boetzer and Pirovano, 2012) is used to fill the GAP between contigs. The genetic elements were predicted by Prokka v1.10 (Seemann, 2014). The ANI was calculated using the OrthoANIu algorithm (Yoon et al., 2017b). The *in silico* DNA-DNA hybridization (isDDH) value between BC051422^T^ and its related type strain was analyzed by Genome-to-Genome Distance Calculator 3.0 (Meier-Kolthoff et al., 2022). All other genome sequences of closely related type strains were obtained from the GenBank database, and the genome sequence of strain BC051422^T^ has been deposited in GenBank/EMBL/DDBJ/PIR (accession number: JAUJUH010000000).

### Physiological and chemotaxonomic analyses

Growth tests were performed at 30 °C on different media, including Luria–Bertani agar, Columbia blood agar, MacConkey agar, Chocolate agar, and Brain Heart Infusion agar (Guangzhou Dijing Microbial Technology Co., Ltd., Guangzhou, China). The growth temperature range was tested at 4, 10, 25, 28, 30, 35, 37, 42, and 50 °C on Columbia blood agar for 48 h. Tolerance to NaCl and pH was detected in test tubes containing 2 ml of LB broth after incubation for 48 h in a thermostatically controlled incubator at different NaCl concentrations (0–10%, w/v, at intervals of 1%) and pH values (4.0–10.0, at intervals of 1.0 pH unit), respectively. Anaerobic growth was performed by incubating cultures on Columbia blood agar for 7 days in an anaerobic bag (bioMerieux). Gram staining was performed using Rapid Gram Stain (Baso Diagnostics, Inc., Zhuhai, China) as per the manufacturer's instructions. The mass spectrometry was conducted on a matrix-assisted laser desorption ionization/time-of-flight mass spectrometry (MALDI-TOF MS; Bruker Daltonik GmbH, Germany) platform using a direct smear of a fresh bacterial colony on the target plate. The bacterial suspension was fixed in glutaraldehyde phosphate buffer overnight and then stained with sodium phosphotungstate. The cell morphology was observed by using a transmission electron microscope (Hitachi TEM System; Tokyo, Japan). The biochemical characterization of strain BC051422^T^ was performed in a microbiochemical tube as per the manufacturer's instructions (Hangzhou Microbial Reagent, Co., Ltd, Hangzhou, China). *E. persicina* GDMCC1.331^T^ was used as a positive control, with 3% (v/v) H_2_O_2_ used to detect the catalytic activity of catalase. The oxidase activity was measured using filter paper soaked with the reagent tetramethyl-p-phenylenediamine. *Erwinia persicina* GDMCC1.331^T^ and *Erwinia sorbitola* J780^T^ were used as control strains for the biochemical analyses. Data for species other than *E. wuhanensis, E. persicina*, and *E. sorbitola* were obtained from the Bacterial Diversity Metadatabase (BacDive) (Reimer et al., 2022). Chemotaxonomic analysis of the BC051422^T^ strain and the reference strain was performed by using the GC platform (Agilent GC 6890), flame ionization detector (FID), and Sherlock Microbial Identification System (Sasser, 1990).

## Results and Discussion

### 16S rRNA gene phylogeny

**Figure 1:**
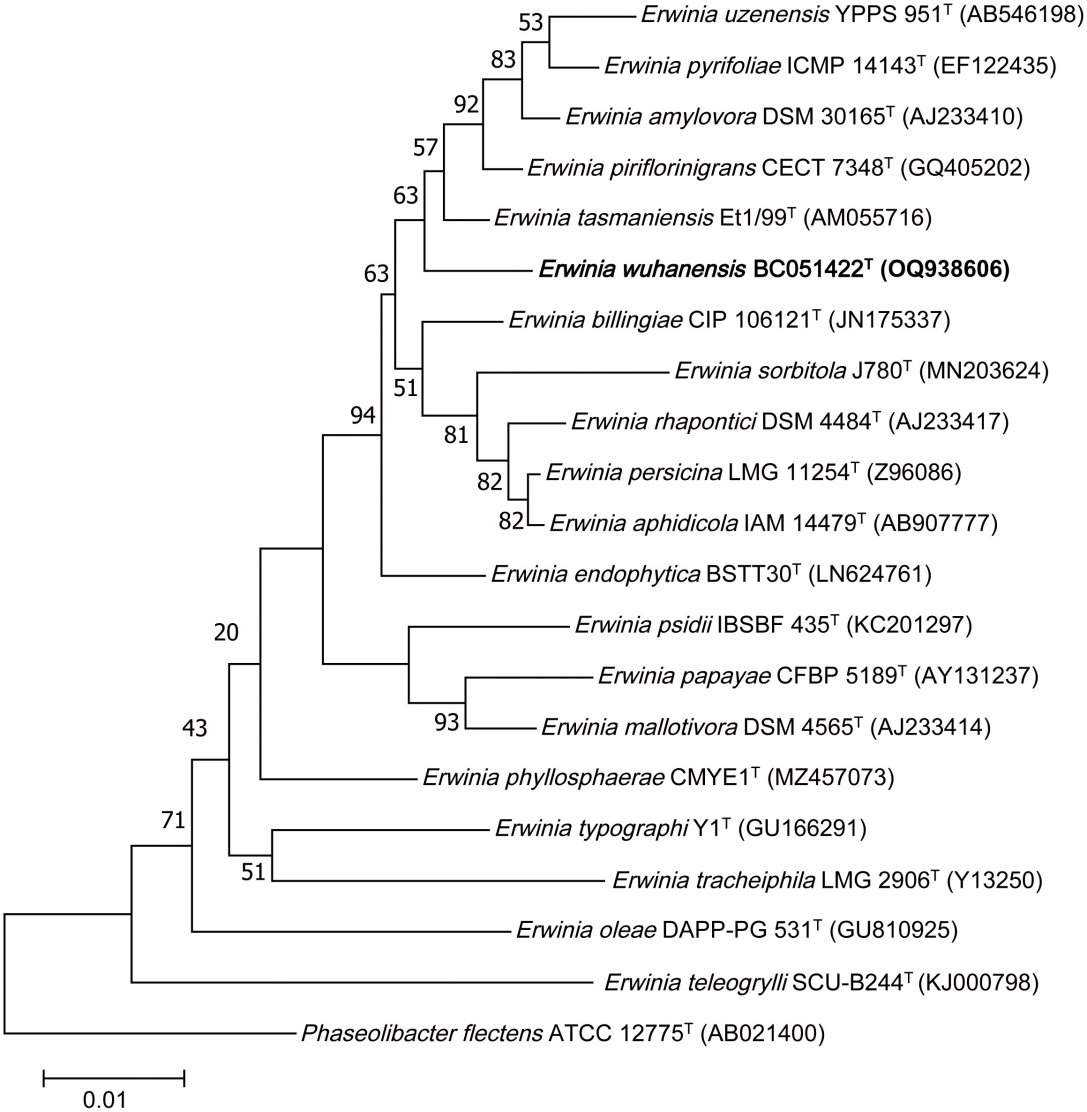
Phylogenetic tree based on the 16S rRNA gene sequences showing the relationship of novel strain BC051422^T^ (bold) and members within genus *Erwinia*. The tree was reconstructed by the neighbor-joining method, and *Phaseolibacter flectens* ATCC 12775^*T*^ (AB021400) was used as an outgroup. Bootstrap values (>50 %) based on 1,000 replicates are shown at branch nodes. T, type strain.

In total, 1,427 contiguous nucleotides of strain BC051422^T^ were sequenced and deposited in GenBank under the accession number OQ938606. The 16S rRNA gene sequence showed the highest similarity to *E. tasmaniensis* Et1/99^T^ (98.74%), followed by *E. piriflorinigrans* CFBP 5888^T^ (98.59%), *E. endophytica* BSTT30^T^ (98.31%), *E. billingiae* CIP 106121^T^ (98.18%), and *Pantoea eucrina* LMG 2781^T^ (97.98%). Similarity to other species of the genera *Erwinia* and *Pantoea* was below 98.00%. A phylogenetic tree constructed using the neighbor-joining method with 1,000 bootstrap replicates showed that strain BC051422^T^ is distinct from previously described species, including the recently published *E. sorbitola* J780^T^ (Figure 1). Additionally, a phylogenetic tree constructed using the maximum likelihood algorithm placed strain BC051422^T^ within the family Erwiniaceae, forming a separate lineage (Supplementary Figure S1). To further validate the evolutionary relationships of the strain BC051422^T^ and closely related species, phylogenetic tree with the method of maximum parsimony was constructed (Supplementary Figure S2).

### Multilocus sequence analysis

**Figure 2:**
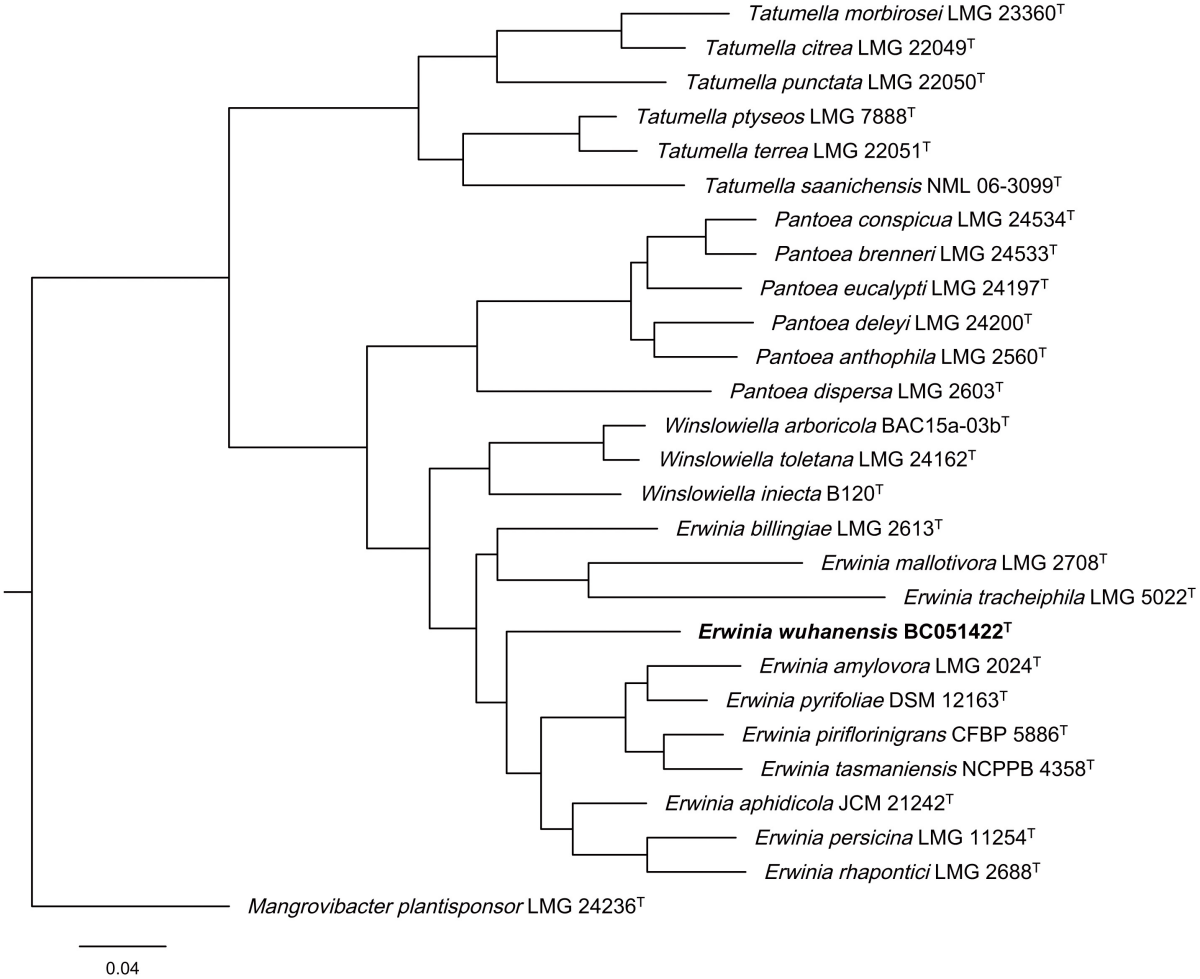
Phylogenetic tree based on concatenated partial *atpD, infB, rpoB*, and *gyrB* gene sequences from the genera *Erwinia, Pantoea, Tatumella*, and *Winslowiella*. The partial genes of strain BC051422^T^ (bold) were retrieved from their whole genome sequences, and the genes of other reference strains were obtained from the GenBank database. The sequences were subjected to PhyloSuite platform according to the guidelines as described. *Mangrovibacter plantisponsor* LMG 24236^T^ was used as outgroup strain.

Multilocus sequence analysis (MLSA) is essential for distinguishing closely related genera such as *Erwinia, Pantoea*, and *Tatumella* (Brady et al., 2008). Phylogenetic analysis based on the concatenated sequences of partial *atpD, infB, rpoB*, and *gyrB* genes revealed that strain BC051422^T^ clusters with type strains of *E. amylovora* LMG2024^T^, *E. aphidicola* JCM21242^T^, *E. tansmaniensis* NCPPB 4358^T^, *E. persicina* LMG11254^T^, and *E. rhapontici* (Figure 2). Sequence accession numbers for all strains used in the MLSA are listed in Supplementary Table S2.

### Genomic characterization

The draft genome of strain BC051422^T^ is 4,596,380 bp in length, assembled into 26 contigs, with an N50 value of 489,674 bp, 200 × coverage, and a GC content of 55.95 mol%. ANI and isDDH values between strain BC051422^T^ and closely related type strains in *Erwiniaceae* ranged 68.75%−83.44% (ANI) and 19.2%−35.5% (isDDH), both well below the species delineation thresholds of 95%−96% (ANI) and 70% (isDDH) (Chun et al., 2018) (Table 1). These results support the classification of strain BC051422^T^ as a novel species within the genus *Erwinia*. A comparative analysis of genomic features among closely related species identified in the phylogenetic tree was performed. The genome size of these species ranges from 3.8Mb to 5.1Mb, with relatively little variation in GC content and gene mumber of tRNA (Supplementary Table S3).

**Table 1:** Average nucleotide identity (ANI), in silico DNA–DNA hybridization (isDDH), and G+C difference values between strain BC051422^T^ and strains of other members of the family *Erwiniaceae*.

**Species**	**Strain**	**ANI value (%)**	**isDDH value (%)**	**G+C difference**
*Erwinia amylovora*	ATCC 15580	77.43	21.8	2.38
*Erwinia aphidicola*	JCM 21238	78.62	22.3	0.70
*Erwinia_billingiae*	TH88	79.69	22.8	0.93
*Erwinia endophytica*	A41C3	78.40	22.2	4.36
*Erwinia mallotivora*	EP60	78.24	21.9	3.66
*Erwinia oleae*	DAPP-PG531	79.19	22.7	1.23
*Erwinia persicina*	NBRC 102418	77.55	21.5	0.55
*Erwinia phyllosphaerae*	CMYE1	83.44	27.2	2.19
*Erwinia piriflorinigrans*	CFBP 5888	77.18	21.4	3.05
*Erwinia psidii*	IBSBF 435	77.80	21.8	4.48
*Erwinia pyrifoliae*	DSM 12163	77.85	21.9	2.58
*Erwinia rhapontici*	CGMCC1.6978	77.40	21.6	2.02
*Erwinia sorbitola*	J780	77.50	22.0	2.79
*Erwinia tasmaniensis*	Et1/99	77.62	21.8	2.57
*Erwinia teleogrylli*	SCU-B244	74.76	20.6	0.63
*Erwinia tracheiphila*	SCR3	76.62	21.4	5.47
*Erwinia typographi*	M043b	80.01	23.3	0.98
*Duffyella gerundensis*	EM595	76.84	21.0	0.68
*Mixta intestinalis*	SRCM103226	76.82	21.0	2.54
*Pantoea agglomerans*	NBRC 102470	76.09	20.7	0.84
*Phaseolibacter flectens*	ATCC 12775	68.75	19.2	11.60
*Tatumella citrea*	DSM 13699	72.25	20.0	6.25
*Wigglesworthia glossinidia*	WGM	70.55	35.5	30.73
*Winslowiella toletana*	DAPP-PG735	77.54	21.3	2.32

### Prediction of pathogenicity

**Figure 3:**
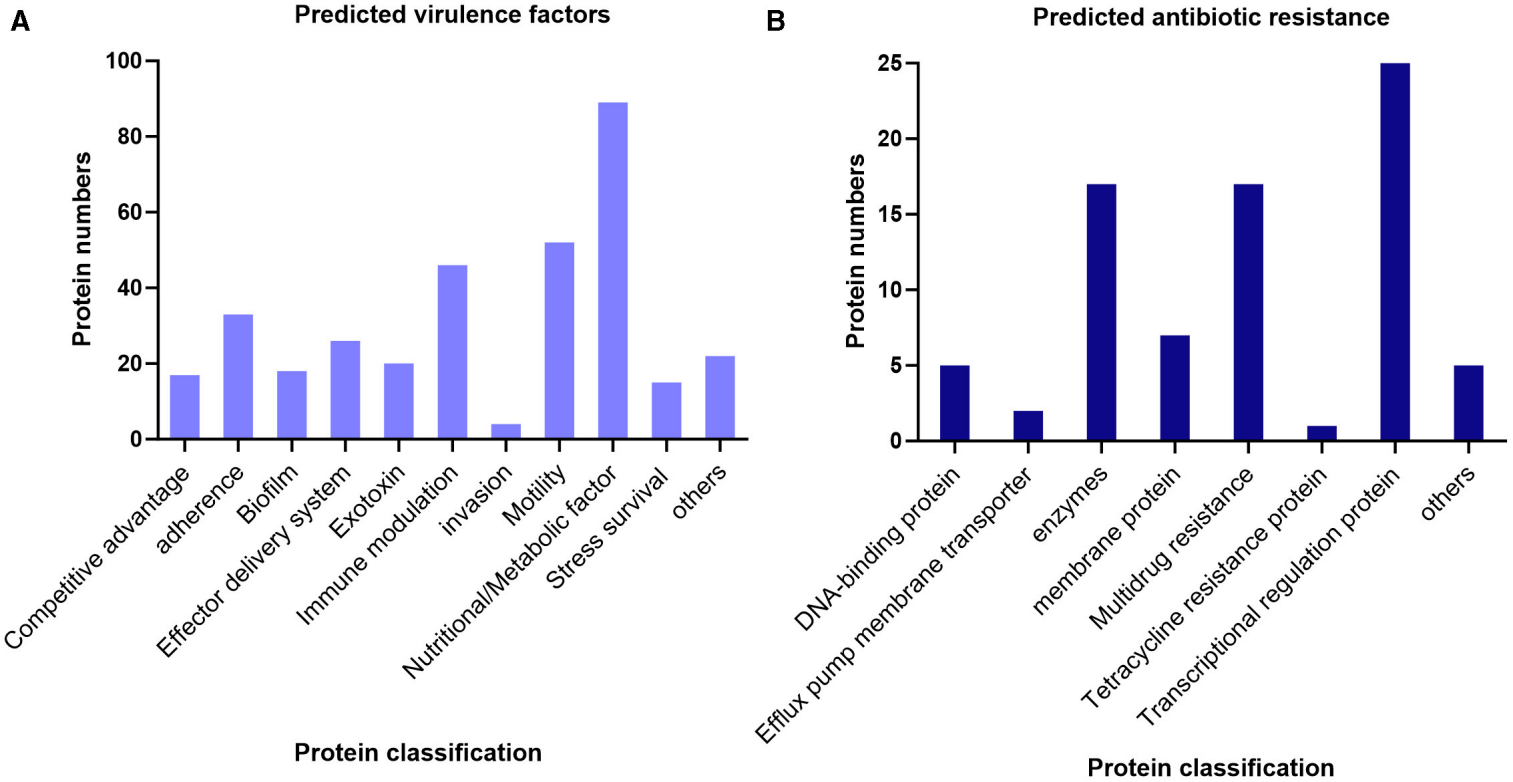
Prediction of pathogenicity of the strain BC051422^T^ through database. **(A)** The virulence factors were predicted through the core dataset of the VFDB. **(B)** The antibiotic resistance was predicted by CARD.

To further explore the pathogenicity of the novel species, an advanced annotation regarding to virulence factors and antibiotic resisrance was conducted. A total of 121 virulence factor terms were predicted through the core dataset of the Virulence Factors of Pathogenic Bacteria (VFDB) (Liu et al., 2022), which contains 342 predicted proteins, accounting for 8.3% of the total proteins (Figure 3A). The database predicted the most nutritional/Metabolic factors, followed by motility and immune modulation related proteins. Adherence and invasion are critical for initial infection of pathogenic organisms, but the predicted invasion-related proteins are in low proportion, suggesting that the novel species may serve as opportunistic pathogen. Additionally, the strain BC051422^T^ was analyzed by the Comprehensive Antibiotic Resistance Database (CARD) (McArthur et al., 2013) and 75 proteins related to antibiotic resistance were predicted (Figure 3B). The antibiotic resistance ontology (ARO) rsmA and CRP, which belong to resistance-nodulation-division (RND) family, were annotated with high identity of matching region (85.25% and 98.57%, respectively).

### Physiology and chemotaxonomy

Strain BC051422^T^ grows well on all tested media, forming visible colonies within 48 h. It grows at 10°C−42°C (optimum: 30°C−35°C), in 0%−8% (w/v) NaCl (optimum: 0%−1% NaCl), and at pH 4.0–9.0 (optimum: pH 7.0), including under anaerobic conditions. Colonies are light yellow, smooth, circular, and 1–2 mm in diameter after 24 h on Columbia blood agar at 30 C (Supplementary Figure S1). Cells are gram-negative rods with peripheral flagella, as observed by microscopy and transmission electron microscopy. A representative mass spectrum was acquired through MALDI-TOF MS (Supplementary Figure S3). Strain BC051422^T^ tested positive for β-galactosidase and nitrate reduction, and negative for arginine dihydrolase, ornithine decarboxylase, lysine decarboxylase, urease, Voges-Proskauer reaction, cytochrome oxidase, and H_2_S production. Acid was produced from glucose, arabinose, and sucrose, but not from mannose, rhamnose, melibiose, or sorbitol (Table 2). These traits differentiate BC051422^T^ from other *Erwinia* species, particularly its ability to ferment sucrose and arabinose, but not mannose, rhamnose, or sorbitol. Detailed results of the aforementioned tests are presented in the species description.

**Table 2:** Biochemical characteristics of strain *E. wuhanensis* BC051422^T^ and type strains of other *Erwinia* species.

**Characteristics**	**1**	**2**	**3**	**4**	**5**	**6**	**7**	**8**
ONPG test	+	+	+	+	–	+	+	–
Nitrate reduction	+	+	+	+	+	+	–	ND
V–P reaction	–	+	–	+	+	+	ND	+
Mannose	–	+	+	+	+	+	–	+
Rhamnose	–	+	+	+	+	–	–	–
Arabinose	+	+	+	+	+	+	+	–
Sucrose	+	+	+	–	+	+	–	+
Melibiose	–	–	–	–	+	+	+	–
Sorbitol	–	+	+	+	–	–	–	+

Taxa: 1, *E. wuhanensis*; 2, *E. persicina*; 3, *E. sorbitola*; 4, *E. billingiae*; 5, *E. rhapontici*; 6, *E. tasmaniensis*; 7, *E. piriflorinigrans*; 8, *E. amylovora*. Data for species other than *E. wuhanensis, E. persicina*, and *E. sorbitola* are obtained from the BacDive database [34]. +, positive; –, negative; ND, not determined.

The major cellular fatty acids of BC051422^T^ were C_16:0_ (31.2%), C_17:0_ cyclo (11.4%), summed feature 3 (C_16:1_ ω7c/C_16:1_ ω6c, 23.9%), and summed feature 8 (C_18:1_ ω7c and/or C_18:1_ ω6c, 10.8%). The fatty acid profile of *E. wuhanensis* and strains of closely related species is presented in Table 3.

**Table 3:** Cellular fatty acid composition (as a percentage of the total) of *Erwinia wuhanensis* and strains of the most closely related species.

**Fatty acids**	**1**	**2**	**3**	**4**	**5**
C12:0	5.8	3.9	4.2	3.4	2.9
C14:0	2.8	6.4	5.4	5.2	5.4
C16:0	31.2	37.7	32.0	36.8	32.1
C17:0 cycle	11.4	NA	13.5	4.9	1.5
C17:0	1.67	NA	NA	0.2	0.3
C18:0	0.6	1.0	NA	0.5	0.5
Summed feature 2^a^	8.6	3.4	9.0	9.2	7.4
Summed feature 3^b^	23.9	36.9	19.5	31.3	35.3
Summed feature 8^c^	10.8	10.8	10.9	7.8	13.4

Taxa: 1, *E. wuhanensis* BC051422^T^; 2, *E. tasmaniensis* LMG 25318; 3, *E. phyllosphaerae* CMYE1^T^; 4, *E. rhapontici* LMG 2688; 5, *E. billingiae* LMG 2613. NA, not available.

^a^Summed feature 2: C_14:0_ 3-OH/iso-C_16:1_I.

^b^Summed feature 3: C_16:1_ ω7c/C_16:1_ ω6c.

^c^Summed feature 8: C18:1 ω7c and/or C18:1 ω6c.

## Conclusions

Based on phylogenetic, genomic, phenotypic, and chemotaxonomic evidence, strain BC051422^T^ represents a novel species of the genus *Erwinia*, for which the name *E. wuhanensis* sp. nov. is proposed.

### Description of *E. wuhanensis* sp. nov.

*E. wuhanensis* (wu.han.en'sis. N.L. fem. adj. *wuhanensis*, referring to Wuhan city, Hubei Province, China, where the type strain was isolated).

Cells are gram-negative, non-spore-forming, facultatively anaerobic rods (1.5–2.5μm), motile with peripheral flagella. Colonies are light yellow, smooth, circular, and 1–2 mm in diameter after 24 h at 30 °C on Columbia blood agar. It grows on LB agar, Columbia blood agar, MacConkey agar, chocolate agar, and brain heart infusion agar. Growth occurs at 10°C−42°C (optimum: 30 C−35 C), in 0%−8% (w/v) NaCl (optimum: 0%−1% NaCl) in brain heart infusion agar, and at pH 4.0–9.0 (optimum: pH 7.0). Positive for catalase, β-galactosidase, aesculin hydrolysis, and nitrate reduction, but negative for cytochrome oxidase, arginine dihydrolase, lysine decarboxylase, ornithine decarboxylase, urease, Voges-Proskauer reaction, and H_2_S production. Ferments glucose, arabinose, and sucrose, but not mannose, rhamnose, melibiose, raffinose, sorbitol, or adonitol. Major cellular fatty acids are C_16:0_, C_17:0_ cyclo, summed feature 3 (C_16:1_ ω7c/C_16:1_ ω6c), and summed feature 8 (C_18:1_ ω7c and/or C_18:1_ ω6c).

The type strain is BC051422^T^, isolated from the blood of a patient at the Central Hospital of Wuhan, Wuhan city, Hubei Province, China, in 2022. The draft genome is 4.6 Mb with a DNA G+C content of 55.95 mol%. Genome and 16S rRNA sequences are available in GenBank/EMBL/DDBJ/PIR under accession numbers JAUJUH010000000 and OQ938606, respectively. The strain is deposited at the Guangdong Microbiology Culture Centre as GDMCC 1.4074^T^ and at the Japan Collection of Microorganisms as JCM 36319^T^.

## Data Availability

The datasets presented in this study can be found in online repositories. The names of the repository/repositories and accession number(s) can be found in the article/Supplementary material.
