# Supplementary material for: Erwinia wuhanensis sp. nov. isolated from human blood
Source: Front Microbiol. 2025 Sep 4;16:1675452. doi: 10.3389/fmicb.2025.1675452 (PMC12443709; doi:10.3389/fmicb.2025.1675452)
Supplement: Supplementary file 1 [file Data_Sheet_1.docx]

***Supplementary Materials***

***Erwinia wuhanensis* sp. nov. isolated from human blood**

Yingmiao Zhang^1,2^, Yu Zhan^1^, Jing Yang^3^*, Zhongxin Lu^1,4^*.


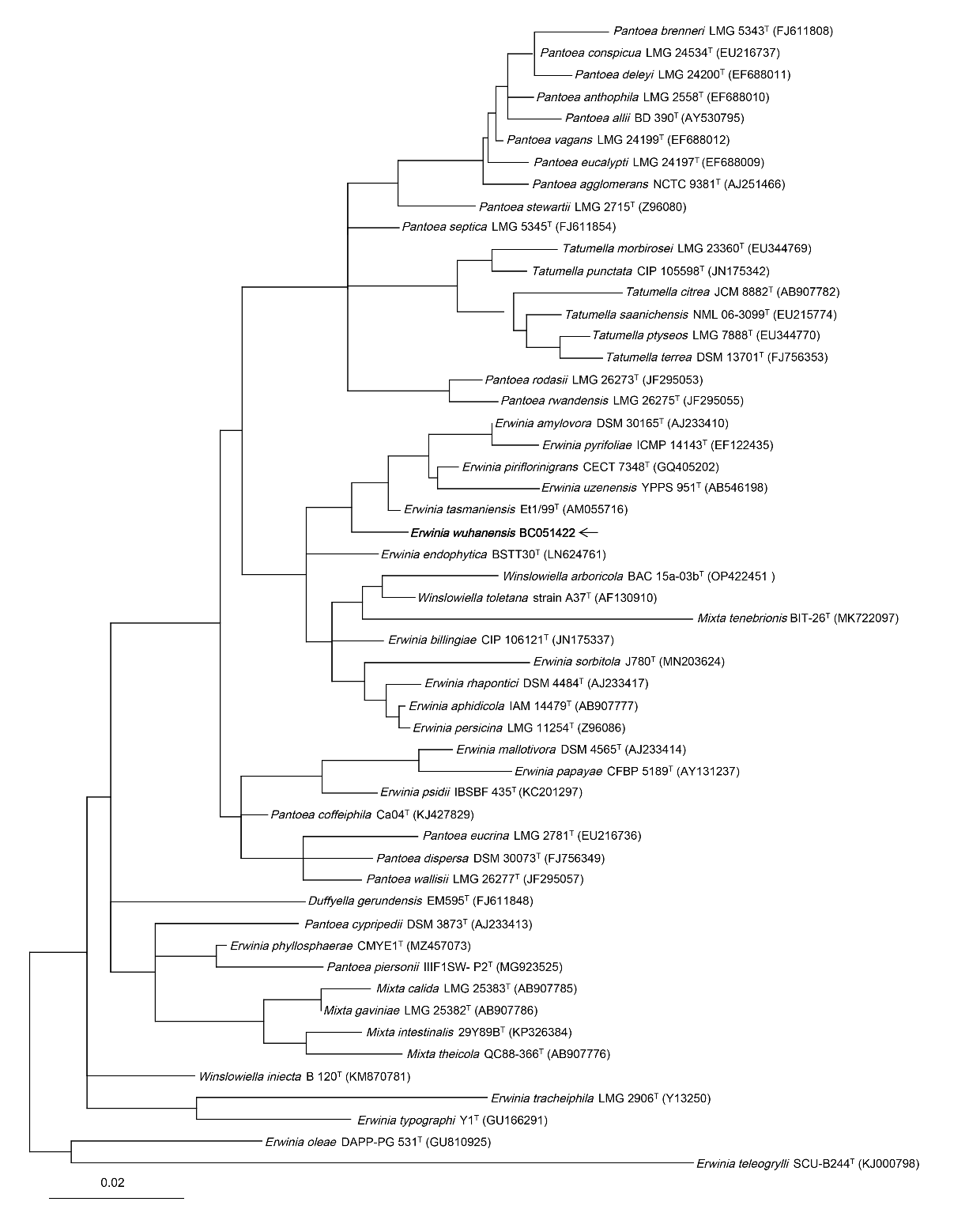


**Supplementary Fig. S1** Phylogenetic tree based on 16S rRNA sequences using maximum-likelihood algorithms with Kimura’s two parameter model indicated that the strain BC051422^T^ was located in family *Erwiniaceae* and formed a separate branch.


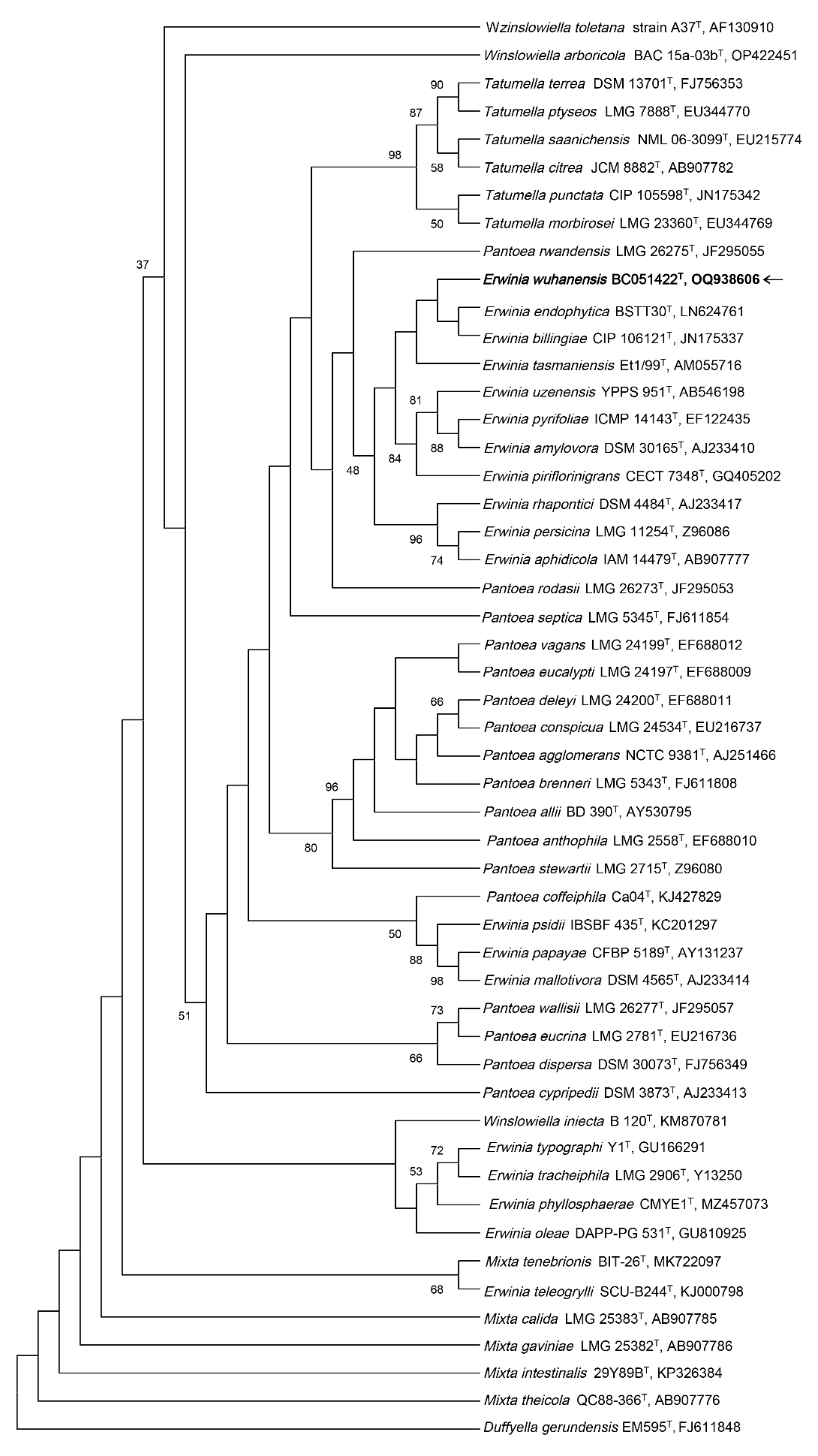


**Supplementary Fig. S2** Phylogenetic tree based on 16S rRNA sequences using maximum parsimony algorithms. .


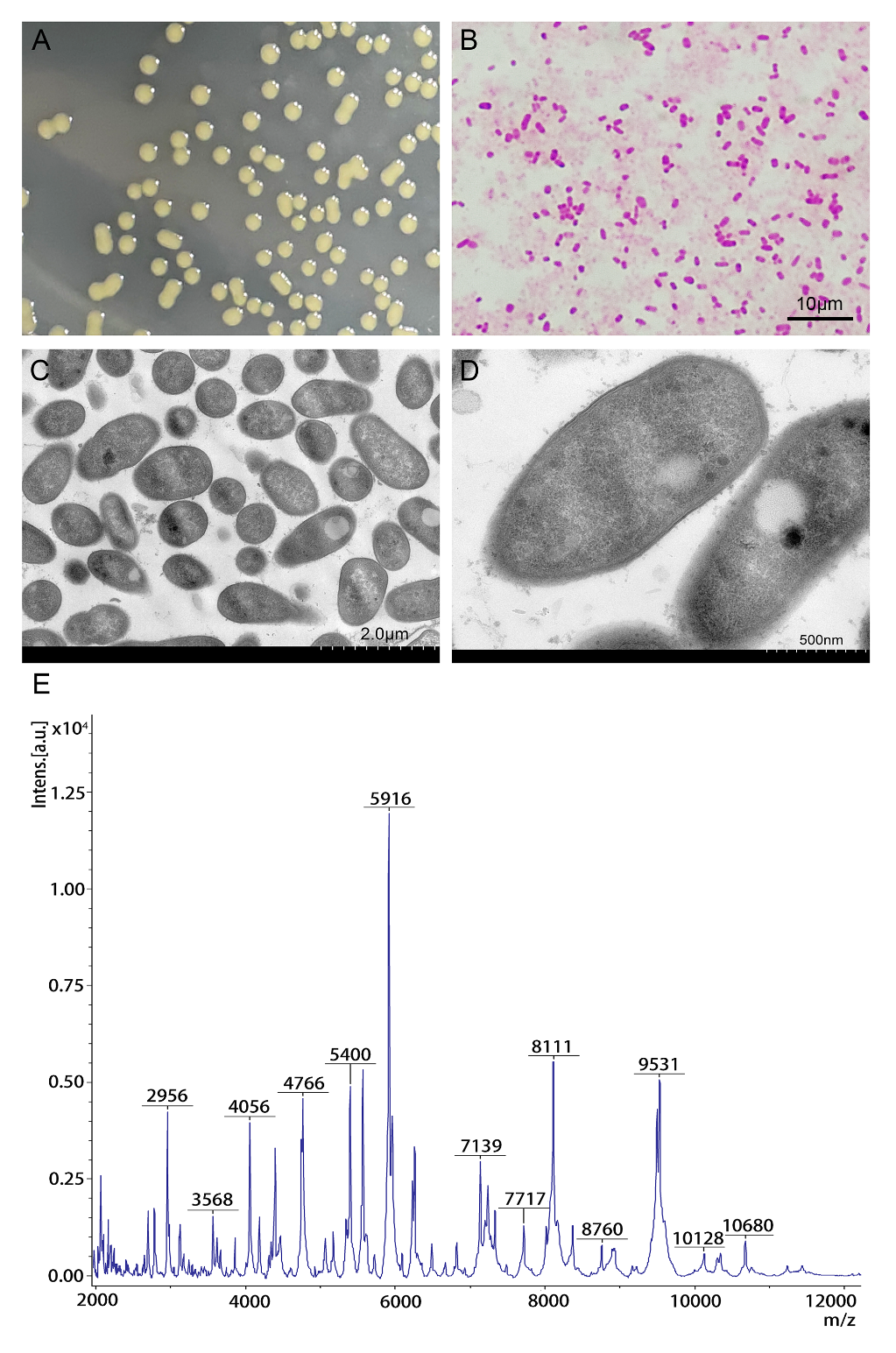


**Supplementary Fig. S3** Morphology and mass spectrum of the novel strain BC051422^T^. Morphology of bacterial colonies after 24 hours of incubation at 30℃ on LB agar (A). Gram straining of the strain (B). Transmission electron microscope of the strain in low (C) and high (D) resolution. Mass spectrum of the strain obtained by MALDI-TOF MS (E).

Table S1. Antimicrobial susceptibility of strain BC051422^T^

| Antibiotics | MICs(mg/L) | Category ^a^ |
| --- | --- | --- |
| Amikacin | ≤ 2 | S |
| Aztreonam | ≤ 1 | S |
| Cefazolin | ≤ 4 | S |
| Cefepime | ≤ 1 | S |
| Cefotetan | ≤ 4 | S |
| Ceftazidime | ≤ 1 | S |
| Ceftriaxone | ≤ 1 | S |
| Ciprofloxacin | ≤ 0.25 | S |
| Gentamicin | ≤ 1 | S |
| Imipenem | ≤ 1 | S |
| Levofloxacin | ≤ 0.25 | S |
| Meropenem | ≤ 0.25 | S |
| Piperacillin | ≤ 4 | S |
| Piperacillin/tazobactam | ≤ 4 | S |
| Tobramycin | ≤ 1 | S |

^a^ S, susceptible; I, intermediate; R, resistant.

**Supplementary** **Table S2** Sequence accession numbers of the strains used for the Multilocus Sequence Analysis.

| **Bacterial species** | **GenBank accession numbers** | | | |
| --- | --- | --- | --- | --- |
|  | ***atpD*** | ***infB*** | ***rpoB*** | ***gyrB*** |
| *Erwinia wuhanensis* BC051422^T^ | WP_12528  9176^r^ | WP_25345  7923^r^ | WP_25345  6284^r^ | WP_25345  6777^r^ |
| *Erwinia amylovora* LMG 2024^T^ | JF311449 | HQ393620 | HQ393632 | HQ393608 |
| *Erwinia aphidicola* JCM 21242^T^ | JF311465 | JF311691 | JF311804 | JF311578 |
| *Erwinia billingiae* LMG 2613^T^ | EU145259 | EU145291 | EU145307 | EU145275 |
| *Erwinia persicina* LMG 11254^T^ | HQ393598 | HQ393622 | HQ393634 | HQ393610 |
| *Erwinia piriflorinigrans* CFBP 5886^T^ | JF311472 | JF311698 | JF311811 | JF311585 |
| *Erwinia pyrifoliae* DSM 12163 ^T^ | HQ393597 | HQ393621 | HQ393633 | HQ393609 |
| *Erwinia mallotivora* LMG 2708^T^ | HQ393589 | HQ393613 | HQ393625 | HQ393601 |
| *Erwinia rhapontici* LMG 2688^T^ | EF988751 | EF988924 | EF989010 | EF988838 |
| *Erwinia tasmaniensis* NCPPB 4358^T^ | HQ393595 | HQ393619 | HQ393631 | HQ393607 |
| *Erwinia tracheiphila* LMG 5022^T^ | HQ393592 | HQ393616 | HQ393628 | HQ393604 |
| *Tatumella morbirosei* LMG 23360 ^T^ | EU344756 | EU344764 | EU344768 | EU344760 |
| *Tatumella ptyseos* LMG 7888 ^T^ | EU145244 | EU145276 | EU145292 | EU145260 |
| *Tatumella punctata* LMG 22050^T^ | EF988716 | EF988889 | EF988975 | EF988803 |
| *Tatumella citrea* LMG 22049^T^ | EF988715 | EF988888 | EF988974 | EF988802 |
| *Tatumella saanichensis*  NML 06-3099^T^ | EU215775 | WP_02968  6332^r^ | EU567306 | WP_02968  5311^r^ |
| *Tatumella terrea* LMG 22051^T^ | EF988717 | EF988890 | EF988976 | EF988804 |
| *Pantoea conspicua* LMG 24534 ^T^ | EU145253 | EU145285 | EU145301 | EU145269 |
| *Pantoea deleyi* LMG 24200^T^ | EF988683 | EF988856 | EF988942 | EF988770 |
| *Pantoea eucalypti* LMG 24197^T^ | EF988675 | EF988848 | EF988934 | EF988762 |
| *Pantoea anthophila* LMG 2560^T^ | EF988726 | EF988899 | EF988985 | EF988813 |
| *Pantoea dispersa* LMG 2603^T^ | EF988731 | EF988904 | EF988990 | FJ617346 |
| *Pantoea brenneri* LMG 24533^T^ | EU145252 | EU145284 | EU145300 | EU145268 |
| *Winslowiella arboricola* BAC15a-03b^T^ | OP414924 | OP414940 | OP414948 | OP414932 |
| *Winslowiella iniecta* B120^T^ | MN089591 | MN089597 | WP_05290  2629^r^ | MN089594 |
| *Winslowiella toletana* LMG 24162^T^ | EU145258 | EU145290 | EU145306 | EU145274 |
| *Mangrovibacter plantisponsor* LMG 24236^T^ | JX424958 | JX425217 | JX425343 | JX425087 |

^r^ Sequences were retrieved from whole genome sequences.

**Supplementary** **Table S3** Genomic features of *Erwinia wuhanensis* and closely related species.

| Bacterial species | Features | | | | |
| --- | --- | --- | --- | --- | --- |
|  | genome size  (base) | GC content  (%) | protein-coding genes | rRNA | tRNA |
| *Erwinia wuhanensis* BC051422^T^ | 4,596,380 | 55.9 | 4,112 | 17 | 79 |
| *Erwinia tasmaniensis* Et1/99 ^T^ | 3,883,467 | 53.5 | 3,622 | 22 | 81 |
| *Erwinia amylovora* LMG 2024 ^T^ | 3,807,395 | 53.5 | 3,255 | 22 | 77 |
| *Erwinia pyrifoliae* DSM 12163 ^T^ | 4,026,286 | 53.5 | 3,714 | 22 | 75 |
| *Erwinia aphidicola* JCM 21242 ^T^ | 5,118,043 | 56.5 | 4,586 | 13 | 79 |
| *Erwinia rhapontici* LMG 2688 ^T^ | 5,057,383 | 54.0 | 4616 | 22 | 82 |
| *Erwinia persicina* CFBP8797 ^T^ | 4,806,044 | 55.5 | 4475 | 6 | 75 |
